# Nocturnal hemoglobin desaturation in chronically transfused adults with sickle cell disease: a retrospective study

**DOI:** 10.1093/jscdis/yoaf003

**Published:** 2025-01-31

**Authors:** Mofiyin A Obadina, Iman Owens, Ada Chang, Vanessa Miller, Jane A Little

**Affiliations:** Division of Hematology, Department of Medicine, University of North Carolina, Chapel Hill, NC 27514, United States; UNC Blood Research Center, University of North Carolina, Chapel Hill, NC 27514, United States; UNC Blood Research Center, University of North Carolina, Chapel Hill, NC 27514, United States; UNC Gillings School of Public Health, University of North Carolina, Chapel Hill, NC 27514, United States; Division of Hematology, Department of Medicine, University of North Carolina, Chapel Hill, NC 27514, United States; UNC Blood Research Center, University of North Carolina, Chapel Hill, NC 27514, United States

**Keywords:** nocturnal Hb desaturation, SCD, chronic transfusion, hypoxemia, sleep disorder

## Abstract

High-risk SCD may be managed with chronic red blood cell exchange transfusions. We examined the prevalence of sleep-associated hypoxemia (≥5 minutes at SpO_2_ ≤88%) or nocturnal Hb desaturation (NHD) in chronically transfused adults with SCD. Of 41 identified participants, 15 (36.6%) had tested positive for NHD at some point prior to enrollment. The median duration of desaturation (SpO_2_ ≤88%) in those that tested positive was 88.3 minutes (IQR 27.9-226.0 minutes). Participants with and without NHD were compared using non-parametric statistical tests. Compared to participants without NHD, those with NHD had higher absolute reticulocyte counts (*P* = .018) and white blood counts (*P* = .05) and tended to be older. They had more anemia (*P* = .11) and higher lactate dehydrogenase levels (*P* = .072). Older age at initiation of chronic red blood cell transfusions associated the strongest with a longer duration of NHD (ρ = 0.4253, *P* = .0067), while prior history of cerebrovascular events associated with a shorter duration of NHD (*P* = .0315). Our results demonstrate that NHD is common in adults being treated with red blood cell exchange for high-risk SCD and associates with laboratory evidence of increased disease activity. Increased awareness of this complication and appropriate screening may provide an additional simple, low-cost, and physiologically relevant treatment intervention, that is oxygen therapy.

## INTRODUCTION

Occult hypoxemia has been reported in pediatric patients with SCD.^1^^-^^6^ It has been described as steady-state, with exercise (post-exertional hypoxemia) and during sleep (nocturnal Hb desaturation, NHD). Sickle cell disease is characterized by abnormal polymerization of the deoxygenated form of Hb S, which is the product of a single amino acid mutation in adult HbA. Polymerization of HbS, and subsequent damage to red blood cells (RBCs), leads to chronic hemolysis, anemia, and vasculopathy, ultimately causing end-organ damage and decreased survival.^7^ Prolonged episodes of Hb desaturation may exacerbate polymerization and drive disease severity. In the pediatric population, NHD has been associated with cerebrovascular, cardiovascular, and genitourinary complications.^1^^,^^8^^-^^12^ The prevalence of NHD, and its sequelae in adults living with SCD, is unknown. Prior studies^4^^,^^6^^,^^13^ showed associations with severe genotypes, increasing age, and disease activity (anemia, hemolysis, and reticulocytosis) but are mostly limited to the pediatric population. A recent prospective study^14^ found NHD per CMS guidelines for oxygen at that time (SpO_2_ ≤88% for >5 minutes) in 9 of 21 adults with high-risk disease (defined by severe genotype, nocturnal symptoms, history of priapism, or excessive reticulocytosis-based on values from a larger registry^15^) and noted associations between NHD and increasing age and anemia.

RBC transfusions, whether as simple or chronic RBC exchange transfusions (RCEs), are effective therapy for acute and chronic complications of SCD. Chronic RCE has been used in stroke prevention, prevention of ACS, prevention of recurrent priapism, prevention of vaso-occlusive pain episodes, and management of pregnancy.^16^ In this study, we attempt to contribute to the adult-specific literature on the prevalence of NHD, using a different high-risk population. At our institution, we recommend NHD testing in adults with concern for sleep-disordered breathing, and other symptoms such as morning headaches, non-restorative sleep, nighttime/morning pain episodes, excessive reticulocytosis, and history of priapism. We hypothesized that NHD was under-recognized in the high-risk subset of adults with SCD who were receiving chronic RCE. The primary objective was to assess the prevalence and duration of NHD on testing, at any time, in this cohort. The secondary objective of the study was to investigate the relationship between NHD and participant characteristics.

## METHODOLOGY

Eligible participants included patients ≥18 years old who had undergone chronic RCE between January 1, 2019 and May 31, 2023. After obtaining institutional review board approval (IRB #21-0274), we performed a retrospective chart review of patients at the University of North Carolina Comprehensive Sickle Cell Center.

We defined chronic transfusion regimen as >2 serial encounters for RCE with a stated indication per the primary hematologist. We defined NHD as SpO_2_ ≤88% for at least 5 minutes of sleep duration, which was the eligibility criteria for oxygen therapy under Center for Medicare and Medicaid Services at the time of evaluation.^17^ We excluded RCEs for acute indications and people with SCD who had NHD testing done during hospital admissions involving acute hypoxemia (ACS, pneumonia). We obtained information about individuals’ RCE regimen (indication, age at initiation) and NHD testing (age at testing, method of testing, and duration of desaturation). We categorized indications for RCE as recurrent ACS, secondary cerebrovascular accident (CVA) prophylaxis, chronic or recurrent pain, pregnancy, recurrent priapism, and other. Indications categorized as “other” were cardiomyopathy, iron overload, leg ulcers, pulmonary hypertension, sickle hepatopathy, and retinopathy. Additional clinical data reviewed were demographics, SCD history, and lab results obtained prior to an encounter for RCE. Labs included complete blood count, Hb electrophoresis, absolute reticulocyte count (ARC), lactate dehydrogenase (LDH), estimated glomerular filtration rate (eGFR), haptoglobin, total bilirubin, C-reactive protein (CRP), and ferritin. All data were obtained from electronic health records. All charts were independently reviewed by 2 members (I.O. and M.A.O.) of the research team.

After assessing data for missingness and assessing continuous variables for normality, frequency (percentage) for categorical variables and median (IQR) for continuous variables were calculated. To compare participants with and without NHD, bivariable analyses were completed using Fisher’s exact test for categorical variables and appropriate non-parametric *t*-tests (Wilcoxon Rank Sum [W-R] or Kruskal Wallis [K-W]) for continuous variables. Non-parametric tests were selected due to the distribution of continuous variables being highly skewed. Comparison by duration of hypoxemia was completed using nonparametric *t*-tests and Spearman’s correlation for categorical and continuous variables, respectively. *P*-values of <.05 were considered statistically significant, and all analyses were performed using Stata 18 (StataCorp LP, College Station, TX).

## RESULTS

We identified 81 adults with SCD who received any exchange transfusion between January 1, 2019 and May 31, 2023. Sixty of these participants were on a chronic RCE regimen as defined in the Methodology section; 41 had a history of NHD testing, and 19 had no NHD testing (11 ordered and pending, 8 never ordered).

Forty-one participants met the criteria of having been on RCE regimen and of having a history of NHD testing. The median age at RCE initiation was 28 years, and the median age at NHD testing was 31 years. Half of the sample (*n* = 21) were female and 97.6% (*n* = 40) of the sample identified as Black or African American. There was no difference in the distribution of sex among the NHD-positive and NHD-negative groups.

Most participants (88%) had Hb SS genotype and were placed on an RCE regimen for recurrent pain episodes (39.0%) or secondary CVA prophylaxis (34.1%). The most common methods of NHD testing were overnight oximetry (43.9%) and polysomnography (39.0%). Twenty-eight (68.3%) participants completed NHD testing after starting an RCE regimen. Of the 41 eligible participants, 31.7% had a diagnosis of obstructive sleep apnea (OSA) and 34.1% had a diagnosis of pulmonary hypertension at the time of NHD testing; ≥15% of data for ARC, total bilirubin, ferritin, LDH, eGFR, CRP, haptoglobin were missing; <3% of data for all other variables in this analysis.

The participants without NHD testing (*n* = 19) did not differ significantly from those who had NHD testing by clinical history and median lab values. They were mostly Hb SS genotype (18/19); the most common indications for an RCE regimen were secondary CVA prophylaxis (9/19) and other indications (7/19). None of these participants had a diagnosis of OSA, and 4/19 had been diagnosed with pulmonary hypertension.

### Prevalence of hypoxemia

Fifteen (36.6%) of eligible participants on RCE regimen tested positive for NHD at any point, and 26 (63.4%) tested negative. Compared to those who were tested negative, those with NHD had a higher median ARC (328.3 × 10^9^/L vs 166.4 × 10^9^/L; *P* = .018) and WBC (13.2 × 10^9^/L vs 10.6 × 10^9^/L; *P* = .050). They tended to be older (median age 36 years vs 26.5 years; *P* = .11), to be more anemic (8.4 g/dL vs 9.1 g/dL; *P* = .063), with higher median LDH levels (496.0 U/L vs 359.0 U/L; *P* = .072). Characteristics of participants are shown in Tables 1 and 2.

**Table 1.** Descriptive summary of participants with SCD on RBC exchange transfusion regimen by NHD test results.

	TOTAL, *N* = 41	NHD positive, *n* = 15	NHD negative, *n* = 26	*P*-value
Female	21 (51.2)	8 (53.3)	13 (50.0)	1.00
Black/African American	40 (97.6)	15 (100.0)	25 (96.2)	1.00
Genotype				.64
SCA (HbSS, HSβ0)	36 (87.8)	14 (93.3)	22 (84.6)	
Variant disease	5 (12.2)	1 (6.7)	4 (15.4)	
Age at NHD testing (year)	31.0 (24.0-39.0)	36.0 (26.0-42.0)	26.5 (22.0-38.0)	.11
Duration of hypoxemia (minutes)	0.9 (0.0-27.9)	0.0 (0.0-0.9)	88.3 (27.9-226.0)	<.001*
Age of exchange initiation (year)	28.0 (18.0-38.0)	32.0 (23.0-39.0)	25.0 (13.0-37.0)	.16
Exchange indication				.26
Recurrent ACS	2 (4.9)	2 (13.3)	0 (0.0)	
Secondary CVA prophylaxis	14 (34.1)	3 (20.0)	11 (42.3)	
Chronic or recurrent pain	16 (39.0)	7 (46.7)	9 (34.6)	
Pregnancy	2 (4.9)	0 (0.0)	2 (7.7)	
Recurrent priapism	3 (7.3)	1 (6.7)	2 (7.7)	
Other	4 (9.8)	2 (13.3)	2 (7.7)	
Method of NHD testing				.026*
Overnight oximetry	18 (43.9)	10 (66.7)	8 (30.8)	
Polysomnography	16 (39.0)	2 (13.3)	14 (53.8)	
Watchpat^®^^18^	7 (17.1)	3 (20.0)	4 (15.4)	
NHD timing				.49
After initiation of exchange regimen	28 (68.3)	9 (60.0)	19 (73.1)	
Before initiation of exchange regimen	13 (31.7)	6 (40.0)	7 (26.9)	
History of				
Pulmonary hypertension	14 (34.1)	6 (40.0)	8 (30.8)	.73
Cerebrovascular accident	19 (46.3)	5 (33.3)	14 (53.8)	.33
Nephropathy	11 (26.8)	6 (40.0)	5 (19.2)	.27
Venous thromboembolism	16 (39.0)	8 (53.3)	8 (30.8)	.19
Priapism	10 (24.4)	4 (26.7)	6 (23.1)	1.00
Opioid use disorder	7 (17.1)	3 (20.0)	4 (15.4)	.69
Prior oxygen use	5 (12.2)	2 (13.3)	3 (11.5)	1.00
Obstructive sleep apnea	13 (31.7)	4 (26.7)	9 (34.6)	.73

Data are presented as median (IQR) for continuous measures and *n* (%) for categorical measures.

* *P*-value <.05; analysis using Fisher’s exact test for categorical measures and Wilcoxon Rank Sum or Kruskal Wallis tests for continuous measures.

**Table 2.** Lab characteristics of participants with SCD on RBC exchange transfusion regimen by NHD test results.

	TOTAL, *N* = 41	NHD positive, *n* = 15	NHD negative, *n* = 26	*P*-value
Hb (g/dL)	8.9 (7.9-9.7)	8.4 (7.1-9.4)	9.1 (8.3-10.4)	.063
Pre-transfusion (Hb S%)	54.3 (40.9-67.8)	55.2 (40.9-76.8)	50.1 (41.3-62.0)	.29
Post-transfusion (Hb S%)	21.2 (15.6-28.2)	26.2 (19.9-29.5)	19.8 (14.7-24.7)	.074
Change in S%	27.8 (23.5-40.6)	24.9 (20.8-53.5)	27.9 (25.7-39.6)	.61
Absolute reticulocyte count (×10^9^/L)	212.0 (120.4-334.2)	328.3 (198.1-379.2)	166.4 (117.8-241.9)	.018*
Lactate dehydrogenase (μ/L)	418.0 (286.0-615.0)	496.0 (318.0-737.0)	359.0 (276.0-533.0)	.072
eGFR (mL/min)	90.0 (88.0-90.0)	90.0 (78.5-90.0)	90.0 (88.0-90.0)	.95
Haptoglobin (mg/dL)	1.0 (1.0-4.0)	1.0 (1.0-1.0)	2.5 (1.0-5.5)	.43
Total bilirubin (mg/dL)	3.3 (1.9-5.3)	3.4 (2.7-5.8)	2.6 (1.4-5.3)	.33
White blood count (×10^9^/L)	11.0 (9.0-14.5)	13.2 (9.8-16.3)	10.6 (8.4-12.6)	.050*
Platelet count (×10^9^/L)	374.0 (277.0-459.0)	390.0 (286.0-502.0)	367.5 (256.0-449.0)	.62

Data are presented as median (IQR) for continuous measures, and *n* (%) for categorical measures.

* *P*-value <0.05; analysis using Wilcoxon Rank Sum test.

### Duration of hypoxemia

**Figure 1.**
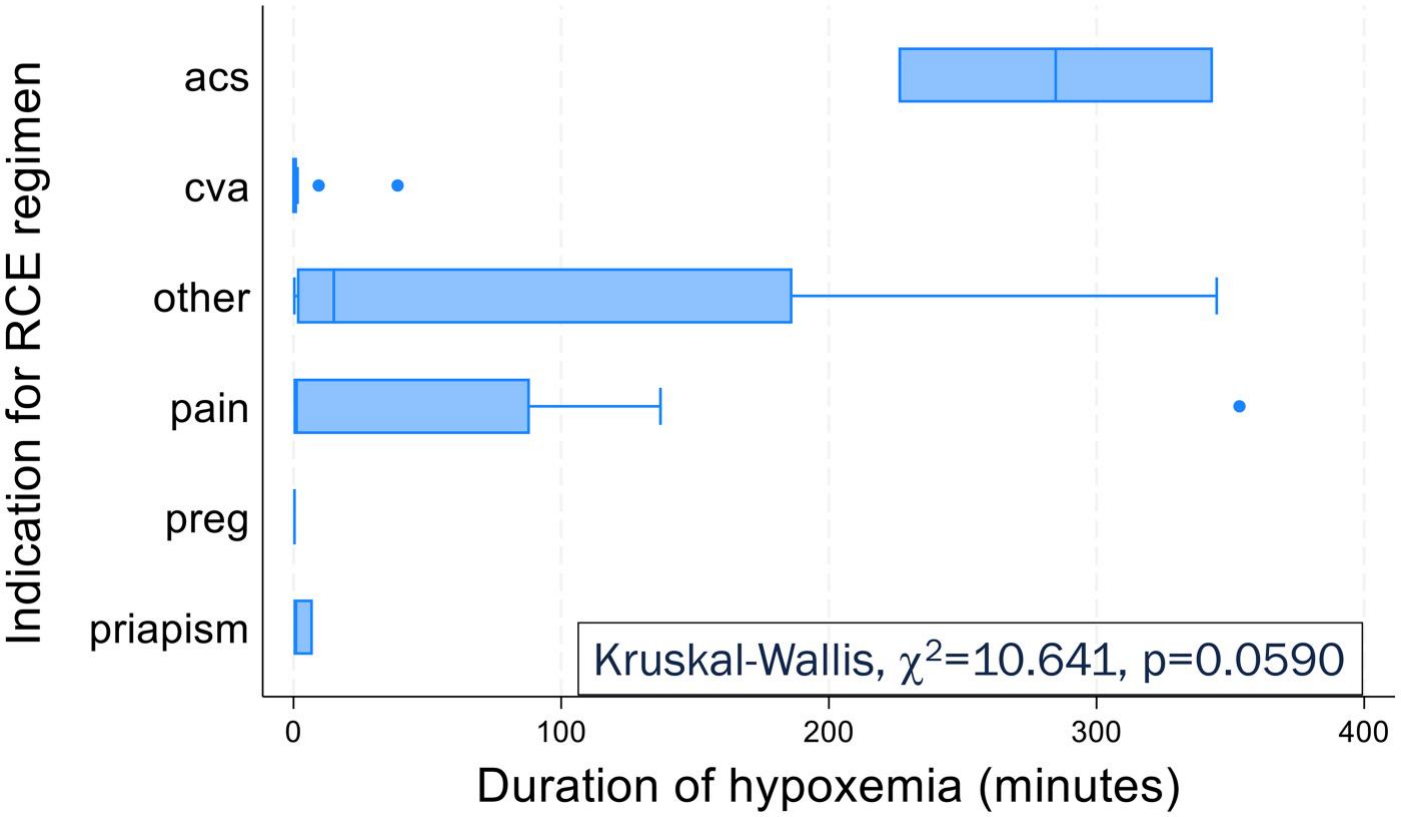
Nocturnal Hb desaturation (NHD) duration by indication for RBC exchange transfusion (RCE) regimen. Participants placed on RCE regimen for secondary cerebrovascular accident prophylaxis (*n* = 14) demonstrated shortest duration of hypoxemia. Indications for RCE regimen: acs, recurrent ACS (*n* = 2); cva, secondary cerebrovascular accident prophylaxis (*n* = 14), pain, chronic or recurrent pain (*n* = 16); preg, pregnancy (*n* = 2); priapism, recurrent priapism (*n* = 3); other, other indications not specified (*n* = 4).

**Figure 2.**
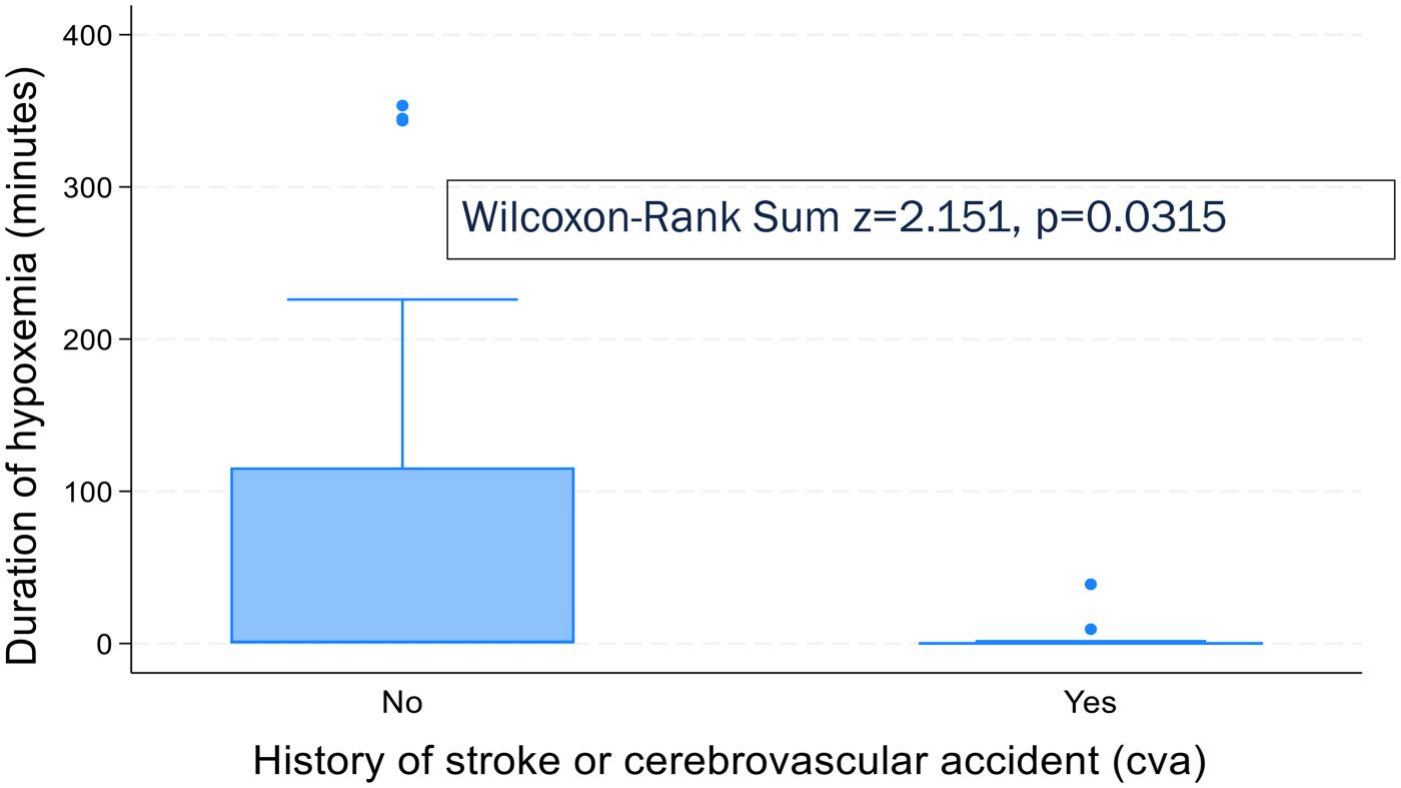
Nocturnal Hb desaturation (NHD) duration by history of stroke or cerebrovascular event. A history of stroke or cerebrovascular accident (CVA) was associated with a statistically significantly shorter duration of hypoxemia.

Using data available for 14/15 participants who tested positive for hypoxemia, the median duration of hypoxemia was 88.3 minutes, with a range of 7.2-353.4 minutes. Three participants had NHD lasting over 5 hours. There was a difference in NHD duration by indication for chronic RCE (K-W, χ^2^ = 10.641, *P* = .0590) as in Figure 1. Unexpectedly, a history of CVA was associated with shorter duration of hypoxemia (W-R, *z* = 2.151, *P* = .0315, Figure 2). NHD duration had strongest correlations (Table 3) with age at RCE initiation (ρ = 0.4253, *P* = .0067), age at NHD testing (ρ = 0.3710, *P* = .019), Hb (ρ= −0.3788, *P* = .0180), post-transfusion Hb S% (ρ = 0.4173, *P* = .0087), ARC (ρ = 0.3645, *P* = .0319), and LDH (ρ = 0.3792, *P* = .0517).

**Table 3.** Strongest bivariable correlations between duration of hypoxemia and participants’ characteristics.

	*n*	Spearman’s ρ	*P* **-value**
Age at exchange initiation	40	0.4253	.0067
Post-transfusion S%	39	0.4173	.0087
Lactate dehydrogenase (LDH)	27	0.3792	.0517
Hb	39	−0.3788	.0180
Age at NHD testing	40	0.3710	.0190
Absolute reticulocyte count (ARC)	35	0.3645	.0319

ρ: Spearman’s correlation coefficient.

### Impact of transfusion on hypoxemia

**Figure 3.**
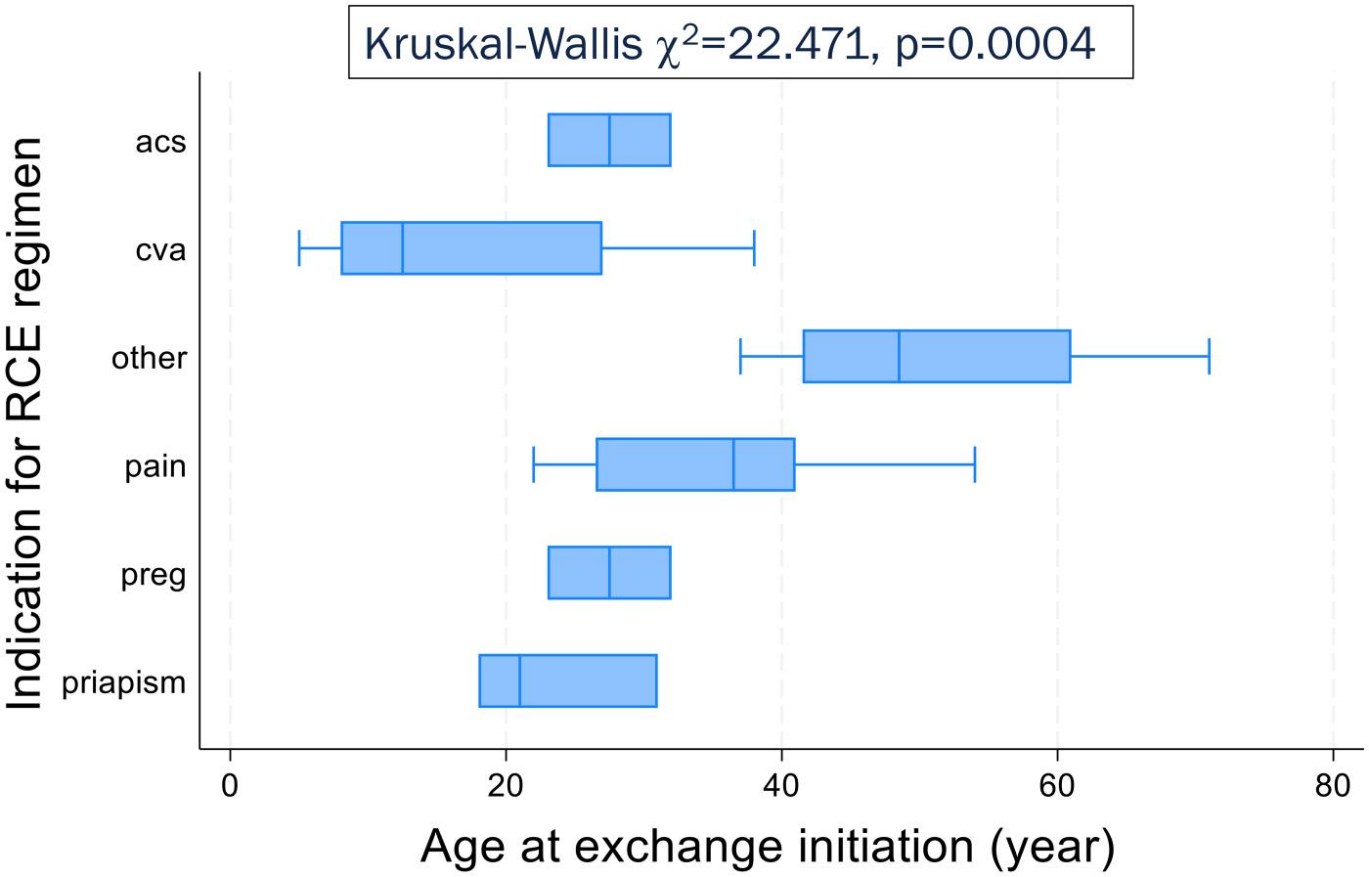
Indication for RBC exchange transfusion (RCE) regimen by age at initiation of regimen. Participants placed on RCE for secondary cerebrovascular accident prophylaxis, who had shorter duration of hypoxemia, were younger on initiation of transfusions, and thus have been on the RCE regimen for longer than the other participants. Indications for RCE regimen: acs, recurrent ACS (*n* = 2); cva, secondary cerebrovascular accident prophylaxis (*n* = 14); pain, chronic or recurrent pain (*n* = 16); preg, pregnancy (*n* = 2), priapism, recurrent priapism (*n* = 3); other, other indications not specified (*n* = 4).

Participants who were started on an RCE regimen earlier in life tended to have negative testing for NHD (W-R, *z* = −1.409, *P* = .1589) and were most likely to be on the regimen for secondary CVA prophylaxis (K-W, χ^2^ = 22.741, *P* = .0004; Figure 3). Additionally, NHD duration showed moderate positive correlation with age at RCE (ρ = 0.4253, *P* = .0067).

Compared to those who had NHD testing at some point before RCE initiation (*n* = 13), participants who had NHD testing after RCE initiation (*n* = 28) tended to have negative NHD results (67.86% vs 53.28%, *P* = .492) and shorter NHD duration (Wilcoxon rank-sum, *z* = −1.276, *P* = .2021).

## DISCUSSION

### Conclusion

In those who completed testing, we found evidence of NHD in over one-third of a high-risk subset of adults with SCD receiving chronic transfusions. The duration of NHD ranged from 7.2 to 353.4 minutes, with a median of 88.3 minutes. The minimum prevalence of NHD in all identified patients undergoing chronic transfusions is 18.5%. As expected, most of the participants had severe genotypes (Hb SS, Hb Sβ0) and were placed on an RCE regimen for recurrent pain and secondary CVA prophylaxis. Positive NHD testing and longer duration of desaturation were associated with greater anemia, reticulocytosis, inflammation, and hemolysis. The age at which RCE regimen was initiated, and therefore the duration on chronic transfusion regimen, demonstrated the strongest correlation with NHD duration. In this cohort, those who were started on RCE regimen at a younger age were being treated for secondary CVA prophylaxis.

### Interpretation, limitation, and next steps

The prevalence of occult hypoxemia in the general adult population with SCD is unknown, and its etiology is not completely understood. To better characterize sleep-associated hypoxemia and explore associations with disease severity, we completed a retrospective study selected for high-risk adults. We selected this enriched population for 2 reasons—to replicate results of earlier studies investigating this phenomenon and to inform providers about potential risk factor for development of occult hypoxemia. The indications for chronic RCE are limited to people with severe disease course, and thus this is a useful population to test our hypothesis that occult hypoxemia is under-recognized in severe SCD.

Our results appear to support this hypothesis. Our previous prospective work^14^ found NHD in 9/21 (43%) adults with severe sickle genotypes, and hypoxemia was associated with greater degree of anemia and markers of inflammation, for example TNFα. As expected, this prevalence is higher than has been reported in studies of unselected adults with SCD. In a study of 43 patients (aged 15-55 years), Stauffer et al.^19^ reported hypoxemia in 16% of adults—this rate was similar to other studies^6^^,^^13^ suggesting a prevalence of 15%-17%. In comparison, the prevalence of nocturnal hypoxemia in the pediatric SCD population has been reported as between 24% and 50%.^5^^,^^20^^,^^21^ However, children with SCD have increased rates of co-morbid OSA compared to the general population, and therefore upper airway obstruction may contribute to the observed hypoxemia. This relationship has not been borne out in adult studies^6^^,^^13^^,^^19^ of sleep-associated hypoxemia, including ours, where the development of NHD is independent of OSA. It should be noted that direct comparisons between many of these studies are limited by variations in their definitions of hypoxemia, which ranged from SpO_2_ ≤88% to 96%.

These data suggest that the etiology of NHD in adults is different from OSA and from intrinsic lung pathology. There is consistency across most studies that anemia associates with occult hypoxemia; however, chronic anemia is not a sufficient explanation.^4^ There seems to be some contribution from increased reticulocytosis and hemolysis, which we replicated in this study. Analyses^19^^,^^22^^-^^24^ of RBC rheology in the context of hypoxia reveal close relationship between hypoxia, decreased deformability, and increased adhesion of RBCs, which may explain the association with hemolysis, that is fragility. The inflammation and endothelial injury associated with hemolysis might also explain the correlation between leukocytosis and NHD in our study. Therefore, the interplay between these factors in the pulmonary vascular environment might predispose patients with severe SCD to development of hypoxemia, via altered oxyhemoglobin dissociation^25^^,^^26^ or development of right-to-left shunts, which have been described^27^^-^^29^ in SCD.

This is reflected in our finding that older age, and therefore longer-standing inflammatory vasculopathy, associates with hypoxemia. This is also supported by the surprising finding that patients who had been receiving exchange transfusions for the longest time virtually had no hypoxemia. Red blood cell exchange transfusion, an evidence-based intervention for secondary stroke prevention, theoretically directly transforms this inflammatory vascular environment^30^ by removing fragile RBCs, reducing hemolysis, and introducing exogenous RBCs with improved oxygen-carrying capacity.

Moreover, this brings up further questions, including whether exchange transfusions can directly prevent the development of nocturnal hypoxemia, and whether oxygen and therapies that modulate RBC health affect the rate of nocturnal hypoxemia. Ultimately, future prospective studies will be needed to determine the impact of these therapies on clinical outcomes and patient survival. While our findings are consistent with previous work, they are limited by the retrospective design and small sample size. Larger, cohort studies will be important in confirming the prevalence and factors involved in NHD. This study’s cross-sectional design also prevents testing for causality; therefore, we are planning a prospective cohort study to test the hypothesis that exchange transfusions mitigate NHD in adults with SCD. In addition, we are working to tease out the relationship between pulmonary right-to-left shunts and hypoxemia in an adult population.

As people with SCD are increasingly living longer, studies of nocturnal hypoxemia provide a potentially actionable opportunity to improve survival and quality of life. While we do not advocate for major changes to screening guidelines, we do hope that providers maintain an increased index of suspicion when evaluating patients with high-risk disease.

No portion of this study was completed with the use of ChatGPT, or other generative artificial intelligence software.
